# Long-term real-world effectiveness and safety of fremanezumab in 1140 patients with migraine and at least 6 months of treatment: third interim analysis of the pan-European PEARL study

**DOI:** 10.1007/s10072-025-08484-1

**Published:** 2025-10-01

**Authors:** Messoud Ashina, Dimos D. Mitsikostas, Faisal Mohammad Amin, Pinar Kokturk, Christoph J. Schankin, Gurdal Sahin, Patricia Pozo-Rosich, Paul J. Dorman, Tomáš Nežádal, Anne Christine Poole, Isabel Pavão Martins, Marja-Liisa Sumelahti, Verena Ramirez Campos, Andrew H. Ahn, Hasan Akcicek, Cristina Tassorelli

**Affiliations:** 1 Department of Neurology, Danish Headache Center, Copenhagen University Hospital - Rigshospitalet, Copenhagen, Denmark; 2 Department of Clinical Medicine, University of Copenhagen, Copenhagen, Denmark; 3 First Department of Neurology, Aeginition Hospital, Medical School, National and Kapodistrian University of Athens, Athens, Greece; 4 Department of Neurorehabilitation/Traumatic Brain Injury, Rigshospitalet, University of Copenhagen, Copenhagen, Denmark; 5 Teva Pharmaceuticals Europe B.V., Haarlem, Netherlands; 6 Department of Neurology, Inselspital, University Hospital Bern, University of Bern, Bern, Switzerland; 7 Department of Clinical Sciences of Lund, Lund University, Skåneuro Neurology Clinic, Lund, Sweden; 8 Neurology Department, Vall d’Hebron University Hospital, Barcelona, Spain; 9 Headache and Neurological Pain Research Group, Vall d’Hebron Research Institute, Departament de Medicina, Universitat Autònoma de Barcelona, Barcelona, Spain; 10 The Newcastle upon Tyne Hospitals NHS Foundation Trust, Newcastle upon Tyne, UK; 11 Institute of Neuropsychiatric Care, Military University Hospital, First Faculty of Medicine, Charles University, Prague, Czech Republic; 12 Private Practice: Oslo Headache Centre, Oslo, Norway; 13 Centro de Estudos Egas Moniz, Faculty of Medicine, University of Lisbon, Lisbon, Portugal; 14 Faculty of Medicine and Health Technology, University of Tampere, Tampere, Finland; 15 Teva Branded Pharmaceutical Products R&D, Inc., West Chester, PA USA; 16 Department of Brain and Behavioral Sciences, University of Pavia, Pavia, Italy; 17 IRCCS C. Mondino Foundation, Pavia, Italy

**Keywords:** Calcitonin gene-related peptide, Chronic, Episodic, Migraine, Real-world data, Real-world evidence

## Abstract

**Introduction:**

Real-world data on the long-term use of fremanezumab for migraine prevention remain limited. This third interim analysis of the PEARL study addresses this gap by investigating the long-term effectiveness, safety, and tolerability of fremanezumab for up to 12 months of treatment.

**Methods:**

PEARL is a 24-month, prospective, observational, Phase 4 study conducted in 11 European countries. Eligible participants were adults (≥ 18 years) diagnosed with chronic or episodic migraine who received subcutaneous fremanezumab (225 mg monthly or 675 mg quarterly) and completed ≥ 6 months of treatment. The primary endpoint was defined as the proportion of participants achieving a ≥ 50% reduction in monthly migraine days (MMD) during the 6-month period following treatment initiation. Secondary endpoints included mean change from baseline to Month 12 in: average MMD, acute migraine medication use, and migraine-related disability scores, as measured by the Migraine Disability Assessment and the 6-item Headache Impact Test. Safety was assessed through the collection of adverse events.

**Results:**

At data cut-off (22 September 2022), 968 of 1140 enrolled participants were included in the effectiveness analysis with 58.5% achieving the primary endpoint. Sustained reductions in MMD, acute medication use, and disability scores were observed over 12 months, and no new safety signals were detected.

**Conclusions:**

Findings from this third interim analysis of PEARL provide compelling evidence for the long-term effectiveness of fremanezumab in a large, real-world patient population. The results support the continued use of fremanezumab as a preventive strategy for migraine and underscore the value of integrating real-world evidence into migraine management.

Trial registration number: EUPAS35111.

## Introduction

Migraine is one of the leading causes of disability worldwide and is associated with a considerable burden on the quality of life and daily function of patients [1]. Although multiple, non-specific oral migraine preventive medications are available to reduce the frequency and severity of migraine attacks, these treatments are often associated with limited efficacy, adverse events, and poor tolerability, resulting in reduced adherence [2–6]. Consequently, patients with suboptimal migraine management frequently cycle through various treatment options, and ultimately rely on acute treatment, which can lead to medication overuse and increased disability [5, 7].

Currently available migraine-specific preventive therapies target the calcitonin gene-related peptide (CGRP) pathway. These therapies include the monoclonal antibodies (mAbs) fremanezumab, eptinezumab, and galcanezumab, which target the CGRP ligand, and erenumab, which targets the CGRP receptor [3, 6, 8]. More recently, small molecule gepants that target the CGRP receptor have been developed for both acute and preventive treatment [9, 10].

Fremanezumab is approved in Europe for the preventive treatment of episodic migraine (EM) and chronic migraine (CM) in adults with ≥ 4 monthly migraine days (MMD), at either monthly (225 mg) or quarterly (675 mg) doses [11]. Randomized controlled trials (RCTs) have demonstrated that fremanezumab effectively reduces headache frequency, severity, and duration in patients with EM and CM [12–18]. Furthermore, fremanezumab’s efficacy, safety, and tolerability have been confirmed across various RCTs that reflect the diverse characteristics of patients with migraine, including those with comorbid major depressive disorder, acute medication overuse, and prior preventive treatment failures [16–18].

Although RCTs provide robust data on treatment efficacy and safety outcomes, they may not fully capture the complexities of patient populations encountered in routine clinical practice. Real-world evidence (RWE), particularly when generated from extended periods, offers insights that are more representative of clinical practice and may reveal safety concerns associated with prolonged drug use [19]. Previous RWE on fremanezumab has been limited to studies conducted in one or two countries, or to retrospective analyses [20–23], and further research is needed to determine its long-term effectiveness, safety, and tolerability. Long-term RWE also aids physicians in understanding patient adherence and persistence with fremanezumab treatment and its impact on migraine-related disability.

The ongoing, Phase 4, Pan-European Real Life (PEARL; EUPAS35111) study represents the largest and most extensive real-world study of fremanezumab to date, enrolling a diverse European population of 1140 individuals with migraine across 87 sites in 11 countries [24]. This study reflects the broad spectrum of patients with migraine by including adults with a range of comorbidities, a wide age range, various concomitant medications, and those who have experienced unsatisfactory responses to previous treatments with other CGRP pathway mAbs. Prior interim analyses of PEARL have already generated promising real-world data on the drug’s effectiveness and tolerability in migraine prevention [25, 26]. Here, we present the results of the third interim analysis, conducted once all participants had completed 6 months of fremanezumab treatment. The additional data available at this cut-off point enable a more comprehensive assessment of the effectiveness, safety, and tolerability of fremanezumab over a longer study period, as well as exploratory outcomes related to treatment adherence, persistence, and overall migraine burden.

## Methods

**Fig. 1:**
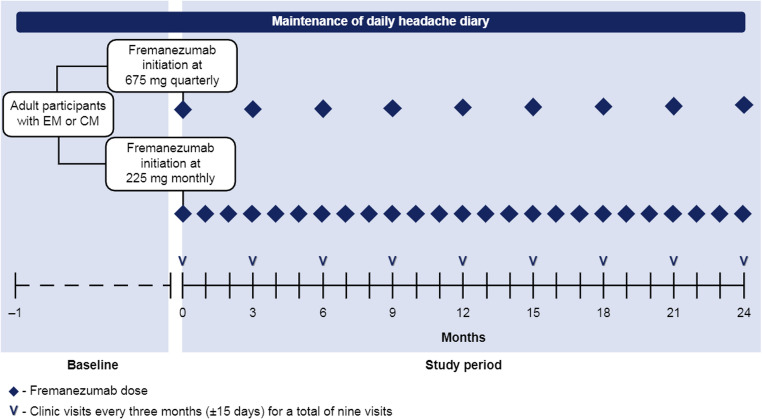
The progress of participants through the PEARL study. Baseline is defined as the 28-day period prior to initiating treatment with fremanezumab; eligible participants have ≥ 21 days of data from this 28-day period in their headache diary. Fremanezumab is initiated within 3 months of the first visit (V0). Ashina M, Mitsikostas DD, Amin FM, Kokturk P, Schankin CJ, Sahin G, Pozo-Rosich P, Dorman PJ, Nežádal T, Poole AC, Martins IP, Sumelahti M-L, Ramirez Campos V, Ahn AH, Lyras L, Tassorelli C. Real-world effectiveness of fremanezumab for the preventive treatment of migraine: Interim analysis of the pan-European, prospective, observational, phase 4 PEARL study. *Cephalalgia.* 2023;43(11):3331024231214987. Copyright @ 2024 by Sage Publications. Reprinted by Permission of Sage Publications. *CM* chronic migraine, *EM* episodic migraine

The flow of participants through the PEARL study is illustrated in Fig. 1. The complete protocol, including all endpoints, participant inclusion and exclusion criteria, and details of the statistical analyses, have been previously published [24]. Here, we describe the methodology used in the third interim analysis.

### Study oversight

The PEARL study protocol was approved by the Independent Ethics Committee/Institutional Review Board in each of the 11 participating European countries, in accordance with local laws and data protection regulations. As a non-interventional, prospective study, PEARL was conducted entirely within the framework of routine clinical practice. Informed consent was obtained from all participants, who agreed to the anonymous recording of their clinical data and retained the right to withdraw their consent at any time during the study.

### Study design

PEARL (EUPAS35111) was a 24-month, prospective, observational, Phase 4 study designed to evaluate the effectiveness, safety, and tolerability of fremanezumab for migraine prevention across 11 European countries (Czech Republic, Denmark, Finland, Greece, Italy, Norway, Portugal, Spain, Sweden, Switzerland, and the United Kingdom).

### Participants

Eligible participants were adults (≥ 18 years) diagnosed with either CM (defined as ≥ 15 headache days/month for > 3 months, ≥ 8 of which meet the International Classification of Headache Disorders criteria for migraine) or EM (≥ 4–14 MMD), who had been prescribed fremanezumab at subcutaneous doses of 225 mg monthly or 675 mg quarterly [24, 27]. Eligible participants also had ≥ 21 days of available headache diary data in the 28 days pre-fremanezumab initiation and were willing to continue to record information in their headache diary throughout the study period. Participants previously treated with another CGRP pathway mAb were also included within this study population as “switch” participants. When switching from another CGRP pathway mAb, participants were recommended to wait until their next scheduled dose before starting fremanezumab.

A total of 1140 participants were enrolled into the PEARL study and followed for a 24-month observational period. The first participant was screened and enrolled in August 2020, and the last participant completed the study in March 2024. The first and second pre-scheduled interim analyses were conducted when 300 and 500 participants, respectively, had completed 6 months of treatment. This third interim analysis was conducted when all enrolled participants had completed 6 months of treatment, with a data cut-off of 22 September 2022.

### Study procedures

As part of routine migraine management, participants maintained a daily headache diary which recorded information on headache frequency, severity, duration, and characteristics, as well as data on concomitant preventive and acute migraine medication. In addition, patient-reported outcome measures (PROMs) were collected using validated headache-related disability assessments. Throughout the 24-month PEARL study period, participants were scheduled for physician visits every 3 months (± 15 days), resulting in a total of nine visits as part of routine care, at the discretion of the treating physician.

### Assessment of outcomes

Baseline data were obtained from headache diary entries recorded during the 28-day period prior to fremanezumab initiation. For each participant, data recorded during the study period were analyzed and compared with baseline data. The primary endpoint was defined as the proportion of participants reaching ≥ 50% reduction from baseline in the monthly average number of MMD during the 6-month period following treatment initiation.

Secondary endpoints, in this interim analysis, assessed at Months 1, 3, 6, 9, and 12, included the mean changes from baseline in the average number of MMD, the monthly number of days of acute migraine medication use, and disability scores as measured by the Migraine Disability Assessment (MIDAS) and the 6-item Headache Impact Test (HIT-6). The MIDAS questionnaire quantifies migraine-related disability over a 3-month recall period (scored as 0–5: little or no disability; 6–10: mild disability; 11–20: moderate disability; and > 20: severe disability) [28]. The HIT-6 assesses the burden of migraine over a 1-month recall period (scored as 36–49, little or no impact; 50–55, some impact; 56–59, substantial impact; and 60–78, severe impact) [29].

Fremanezumab treatment adherence and persistence were also assessed as secondary endpoints. Adherence was defined as participants who took their prescribed dose within ± 5 days of the scheduled monthly or quarterly dosing regimen. Non-adherence was defined as participants who took their prescribed dose ± 5 days outside of their scheduled dose regimen, from the time of the first dose occurring outside such an interval, even if they were adherent at following timepoints. An alternative measure of adherence was participants who took their prescribed dose within ± 5 days of the scheduled monthly or quarterly dosing regimen, per injection. Persistence with fremanezumab was measured by continued administration (unless required to discontinue by their healthcare professional due to a successful course of treatment or local reimbursement conditions).

Exploratory endpoints in this interim analysis included the change from baseline in mean peak headache severity of remaining migraine attacks (measured on an 11-point numerical rating scale [NRS]; 0 = no pain; 10 = worst pain possible) and mean monthly duration of remaining migraine attacks (measured through headache diary entries). The effectiveness of switching from another CGRP pathway mAb to fremanezumab, defined as the proportion of participants previously treated with another CGRP pathway mAb achieving ≥ 50% reduction from baseline in MMD during the 6 months after fremanezumab initiation, was also explored.

Safety was evaluated based on the documentation of adverse events (AEs) reported during routine clinical practice [24].

### Statistical analyses

Safety was analyzed in the safety analysis set (SAS), which included all participants enrolled in the study. Effectiveness outcomes were analyzed in the full analysis set (FAS) using PROMs from participant diaries and other validated headache-related disability tools specified above. The FAS included all enrolled participants who had ≥ 10 days of recorded data for the primary endpoint. Reasons for participant exclusion from the FAS included: missing baseline data; discontinuation due to protocol deviation; insufficient migraine days in the baseline or observation period; and/or no diary data.

All variables of the PEARL study are summarized descriptively. Continuous variables were analyzed with descriptive statistics for their actual values and changes from baseline to each visit; for categorical variables, frequency and percentage are provided. Data for primary, secondary, and exploratory endpoints are presented as both mean values across timepoints and as mean changes from baseline. The mean value is the mean for all participants at a given timepoint, whereas the mean change from baseline measures the change between two timepoints for each individual participant. Therefore, the number of participants for the mean change is typically lower than for the change from baseline, as data must be available for individual participants at both timepoints.

The number of participants prematurely discontinuing this study and the number of participants that had not yet reached the relevant observation timepoint, as well as delays in data being entered into the electronic data capture system, contributed to a drop in participant numbers over time. Due to the real-world nature of the study and reliance on headache diaries, not all data were available for every participant for each timepoint and/or endpoint. In these instances, participants with missing data were excluded from the relevant timepoint or endpoint analysis.

## Results

### Participant disposition and baseline characteristics

**Fig. 2:**
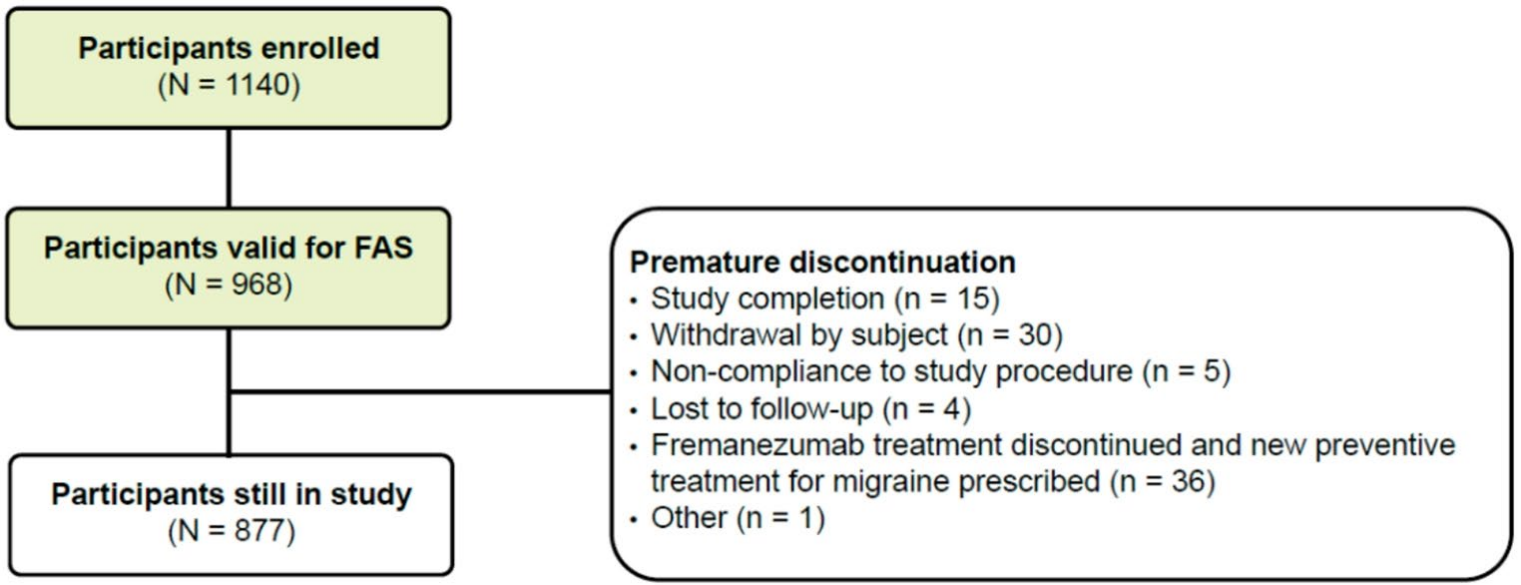
Participant inclusion process. Data cut-off date: 22 September 2022. *FAS* full analysis set

Of 1140 participants enrolled in PEARL and included in the SAS, 968 had ≥ 10 days of recorded data for the primary endpoint and were included in the FAS (Fig. 2). At data cut-off, 877 (90.6%) participants were still enrolled in the study. The most frequent reasons for discontinuation included study completion (n = 15), study withdrawal (n = 30), and changing to a new preventive migraine treatment (n = 36) (Fig. 2).

Baseline characteristics are shown in Table 1. The mean age was 46.5 years (standard deviation [SD], 11.8), and most participants were female (n = 845; 87.3%), and white (n = 919; 94.9%). Two thirds of participants had a diagnosis of CM (n = 648; 66.9%). A total of 865 participants received fremanezumab monthly (89.4%), 60 received quarterly (6.2%), and 43 received ‘other’ (4.4%), which included monthly and quarterly dosing. The mean time from date of diagnosis to fremanezumab initiation was 17.3 (SD, 12.5) years (EM, 17.6 [SD, 12.2]; CM, 17.1 [SD, 12.7]). Depression (n = 129; 13.3%) and insomnia (n = 55; 5.7%), which were categorized under psychiatric disorders (n = 263; 27.2%) were among common comorbid disorders, followed by musculoskeletal and connective tissue disorders (n = 159; 16.4%); vascular disorders (n = 137; 14.2%) such as hypertension (n = 103; 10.6%); gastrointestinal disorders (n = 132; 13.6%); metabolism and nutritional disorders (n = 124; 12.8%); and endocrine disorders (n = 111; 11.5%) such as hypothyroidism (n = 71; 7.3%). In the 5 years prior to informed consent, the most frequently used preventive migraine medication classes were anticonvulsants (n = 665; 68.7%), beta-blockers (n = 590; 61.0%), and tricyclic antidepressants (n = 552; 57.0%). Other CGRP pathway mAbs used for the preventive treatment of migraine during the 5 years prior to informed consent were erenumab (n = 85; 8.8%) and galcanezumab (n = 4; 0.4%) (Table 2).

**Table 1:** Participant demographic and baseline characteristics

Characteristic	FAS^a^ (N = 968)
Age (years), mean (SD)	46.5 (11.8)
Female, n (%)	845 (87.3)
Race, n (%)	
White	919 (94.9)
Asian	1 (0.1)
Not reported	48 (5.0)
BMI (kg/m^2^), mean (SD)^b^	24.5 (4.5)
Migraine type, n (%)	
EM	320 (33.1)
CM	648 (66.9)
Common comorbidities, n (%)^b,c,d,e^	
*Psychiatric disorders*	263 (27.2)
Depression	129 (13.3)
Insomnia	55 (5.7)
*Musculoskeletal and connective tissue disorders*	159 (16.4)
*Vascular disorders*	137 (14.2)
Hypertension	103 (10.6)
*Gastrointestinal disorders*	132 (13.6)
*Metabolism and nutrition disorders*	124 (12.8)
*Endocrine disorders*	111 (11.5)
Hypothyroidism	71 (7.3)
Time from initial migraine onset date to fremanezumab initiation (years), mean (SD)	
Total	27.4 (13.3)
EM	27.6 (12.3)
CM	27.3 (13.8)
Time from date of official diagnosis to fremanezumab initiation (years), mean (SD)	
Total	17.3 (12.5)
EM	17.6 (12.2)
CM	17.1 (12.7)
Concomitant medication, n (%)	
Any concomitant medication	816 (84.3)
Concomitant migraine medication	626 (64.7)
Concomitant acute migraine medication	497 (51.3)
Concomitant preventive migraine medication	321 (33.2)
Other concomitant medication	641 (66.2)
Fremanezumab dosage, n (%)	
Only monthly	865 (89.4)
Only quarterly	60 (6.2)
Monthly and quarterly	43 (4.4)

^a^Includes enrolled participants with ≥10 days of diary entry data post-treatment initiation.

^b^Total participants with medical history (n = 750).

^c^Includes system organ class reported in ≥10% of the study population.

^d^Includes specific disorders reported in ≥5% of the study population.

^e^Some participants can have several different comorbidities and thus, the numbers do not sum up to the total number of participants.

*BMI* body mass index, *CM* chronic migraine, *EM* episodic migraine, *FAS* full analysis set, *SD* standard deviation

**Table 2:** Past preventive migraine therapy

	Total (N= 968)	EM (n = 320)	CM (n = 648)
Past preventive migraine therapy, n (%)^a^			
Anticonvulsants	665 (68.7)	256 (80.0)	409 (63.1)
Beta-blockers	590 (61.0)	187 (58.4)	403 (62.2)
Tricyclic antidepressants	552 (57.0)	163 (50.9)	389 (60.0)
Calcium channel blocker	371 (38.3)	168 (52.5)	203 (31.3)
Onabotulinumtoxin A	344 (35.5)	37 (11.6)	307 (47.4)
Angiotensin II receptor antagonist	149 (15.4)	10 (3.1)	139 (21.5)
Valproic acid	135 (13.9)	48 (15.0)	87 (13.4)
Erenumab	85 (8.8)	21 (6.6)	64 (9.9)
Galcanezumab	4 (0.4)	1 (0.3)	3 (0.5)

^a^During the 5 years prior to informed consent.

*CM* chronic migraine, *EM* episodic migraine

### Primary endpoint

**Fig. 3:**
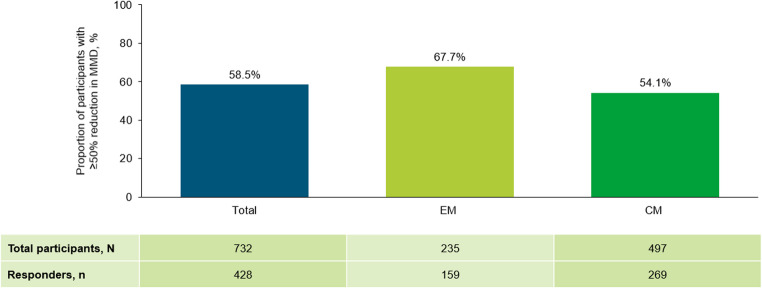
Proportion of participants reaching ≥ 50% reduction in MMD during the 6 months after fremanezumab initiation. At data cut-off, not all data for this endpoint were available and participants with missing data have been excluded. *CM* chronic migraine, *EM* episodic migraine, *MMD* monthly migraine days

Of the 732 participants with available data, 428 (58.5%) achieved a ≥ 50% reduction in MMD during the 6-month period after fremanezumab initiation (Fig. 3). A ≥ 50% reduction in MMD was achieved in 67.7% (n = 159) of participants with EM and 54.1% (n = 269) of participants with CM (Fig. 3).

### Secondary endpoints

#### Reduction and change from baseline in MMD

**Fig. 4:**
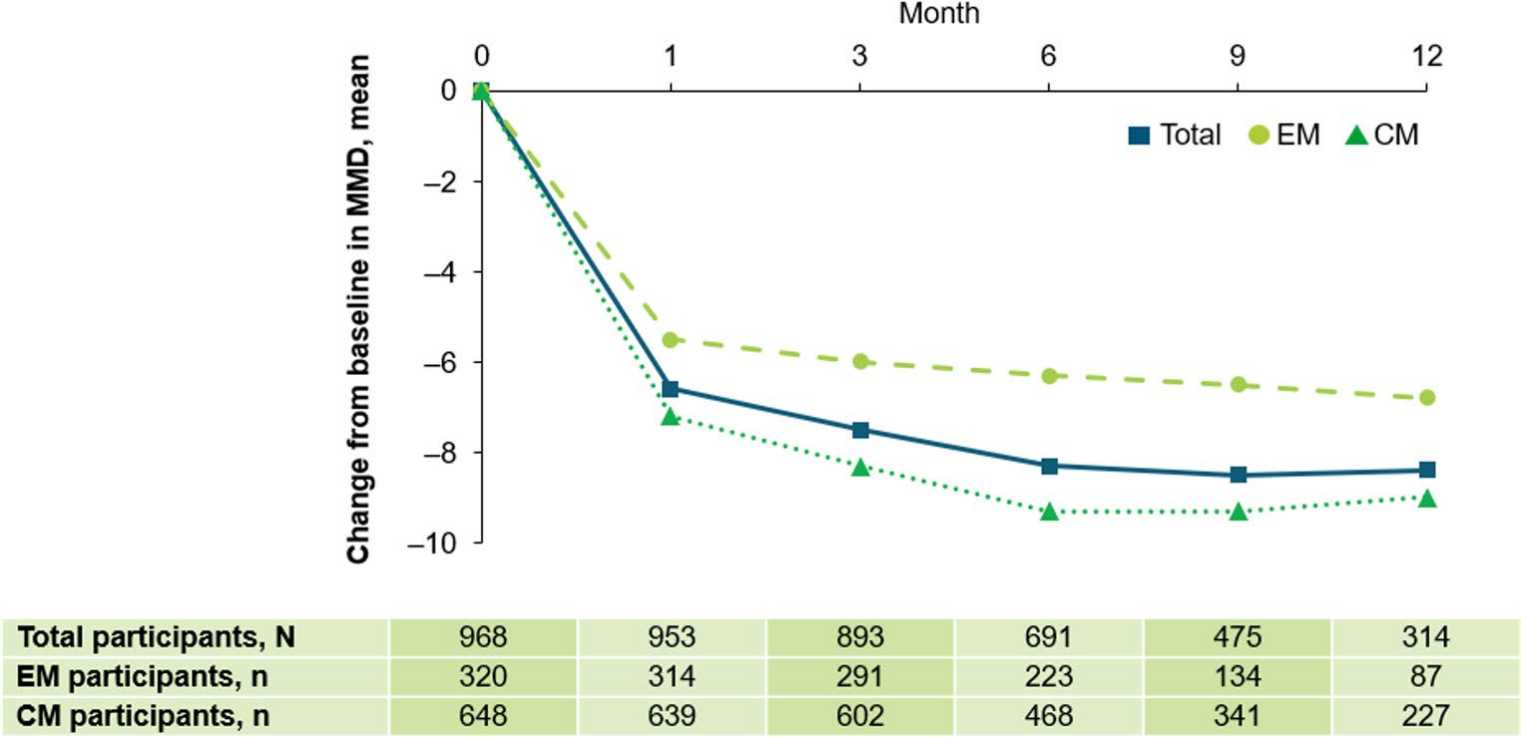
Mean change from baseline in MMD at Months 1, 3, 6, 9, and 12. At data cut-off, not all data for this endpoint were available and participants with missing data have been excluded. The number of participants prematurely discontinuing this study and the number of participants that had not yet reached the relevant observation timepoint, as well as delays in data being entered into the electronic data capture system, contributed to a drop in participant numbers over time. *CM* chronic migraine, *EM* episodic migraine, *MMD* monthly migraine days

**Fig. 5:**
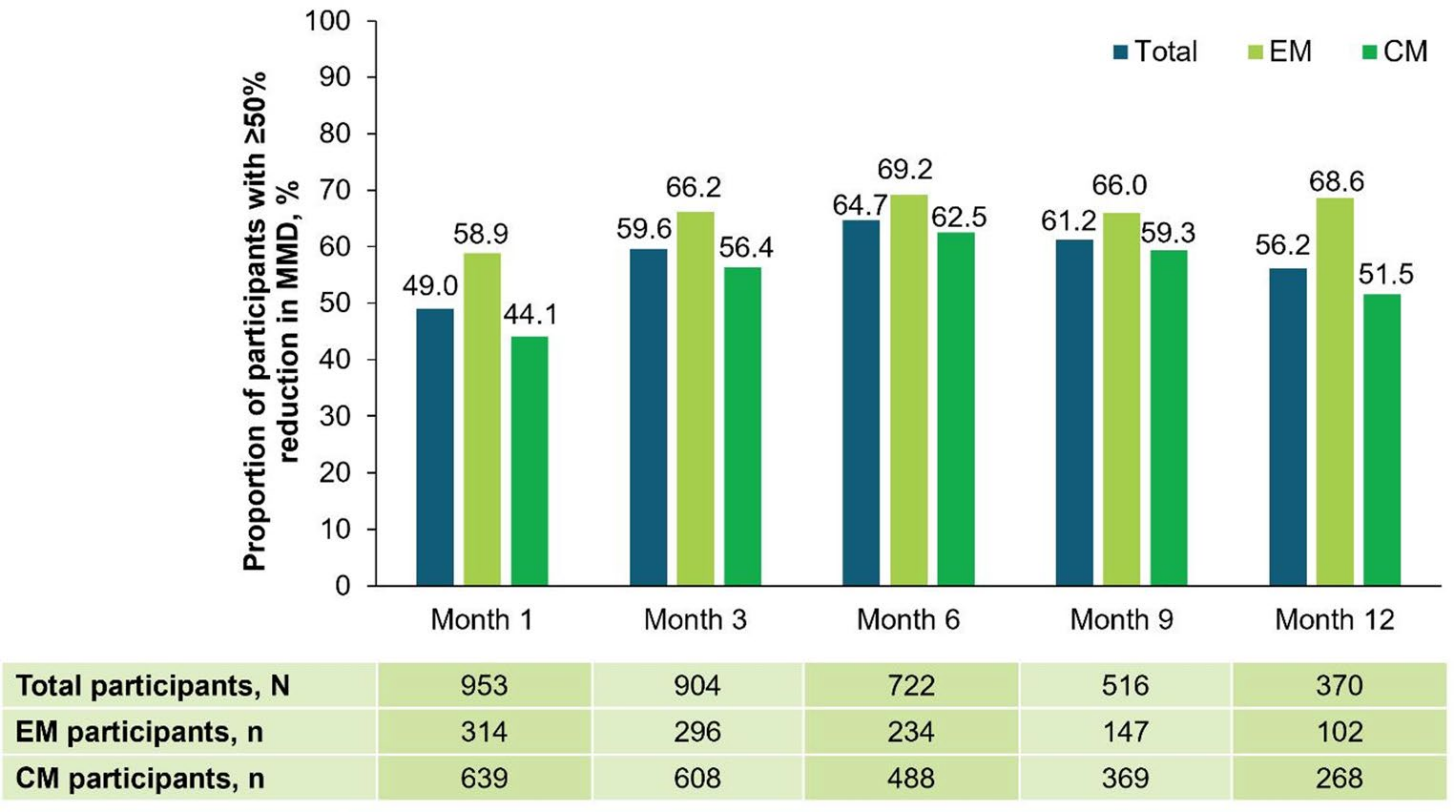
Proportion of participants reaching ≥ 50% reduction in MMD at Months 1, 3, 6, 9, and 12 after fremanezumab initiation. At data cut-off, not all data for this endpoint were available and participants with missing data have been excluded. The number of participants prematurely discontinuing this study and the number of participants that had not yet reached the relevant observation timepoint, as well as delays in data being entered into the electronic data capture system, contributed to a drop in participant numbers over time. *CM* chronic migraine, *EM* episodic migraine, *MMD* monthly migraine days

At baseline, the mean number of MMD for all participants (N = 968) was 14.6. Following fremanezumab initiation, this decreased to 7.9, 7.0, 6.1, 6.1, and 6.1 at Months 1, 3, 6, 9, and 12, respectively. In participants with EM, MMD reduced from 10.3 at baseline to 4.8, 4.3, 3.7, 3.4, and 3.1 at Months 1, 3, 6, 9, and 12, respectively. In participants with CM, MMD decreased from 16.6 at baseline to 9.5, 8.3, 7.3, 7.2, and 7.3 at Months 1, 3, 6, 9, and 12, respectively. For all participants, the mean change from baseline in MMD at 6 and 12 months was − 8.3 (EM, − 6.3; CM, − 9.3) and –8.4 (EM, − 6.8; CM, − 9.0; Fig. 4), respectively. The proportion of participants that achieved a ≥ 50% reduction in MMD was 49.0% (n = 467) at Month 1, 59.6% (n = 539) at Month 3, 64.7% (n = 467) at Month 6, 61.2% (n = 316) at Month 9, and 56.2% (n = 208) at Month 12 (Fig. 5).

#### Acute migraine medication use

**Fig. 6:**
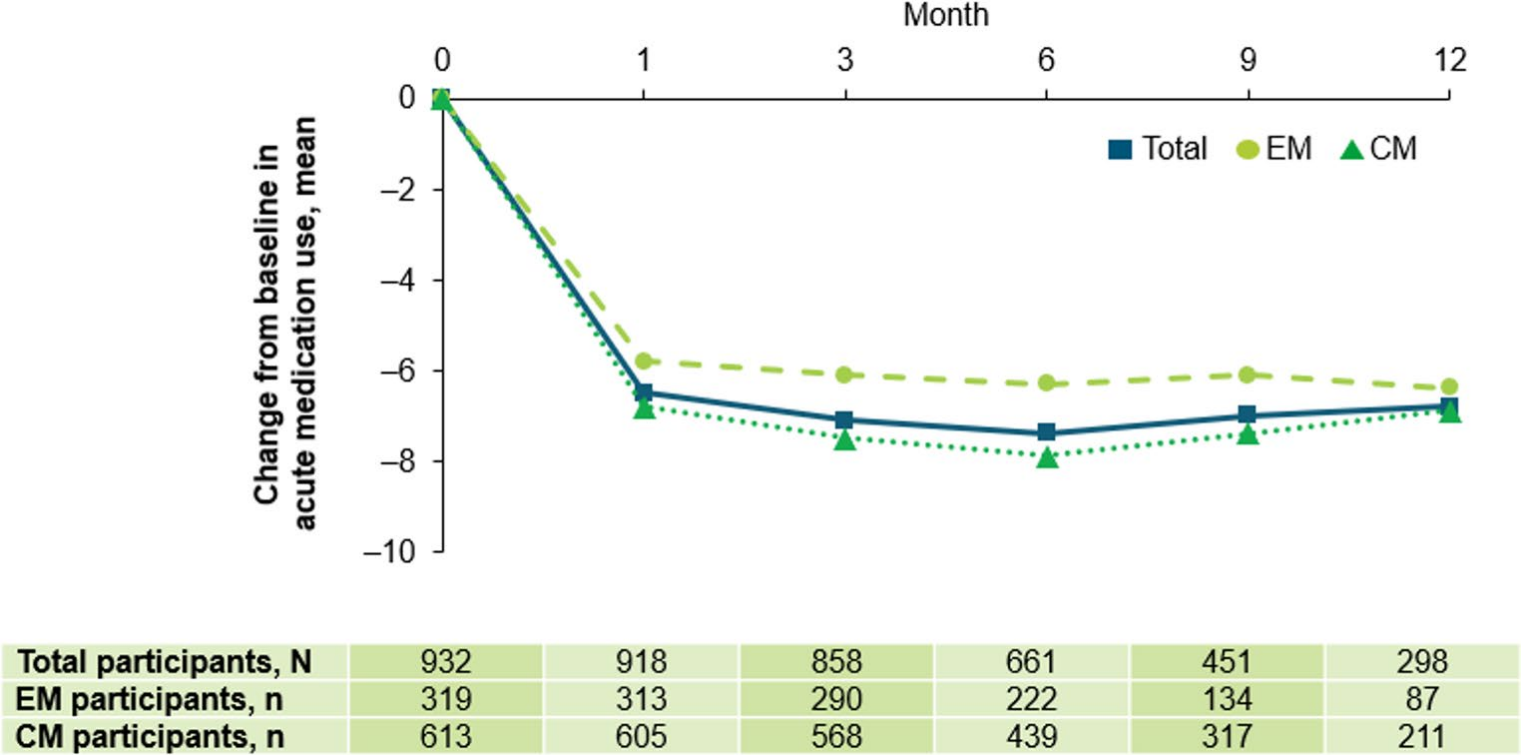
Mean change from baseline in acute medication use at Months 1, 3, 6, 9, and 12. At data cut-off, not all data for this endpoint were available and participants with missing data have been excluded. The number of participants prematurely discontinuing this study and the number of participants that had not yet reached the relevant observation timepoint, as well as delays in data being entered into the electronic data capture system, contributed to a drop in participant numbers over time. *CM* chronic migraine, *EM* episodic migraine

Approximately half of all enrolled participants (n = 497; 51.3%) used concomitant acute migraine medication. At baseline, the monthly average number of days with acute medication use was 11.9 for all participants (EM: 9.7, CM: 13.0). The average number of days with acute medication use decreased to 5.2, 4.7, 4.1, 4.2, and 4.2 at Months 1, 3, 6, 9, and 12, respectively. The change from baseline in acute medication use following fremanezumab initiation remained consistent, from ‒6.5 at Month 1 to ‒6.8 at Month 12 in the total participant population (Fig. 6).

#### Migraine related disability

**Fig. 7:**
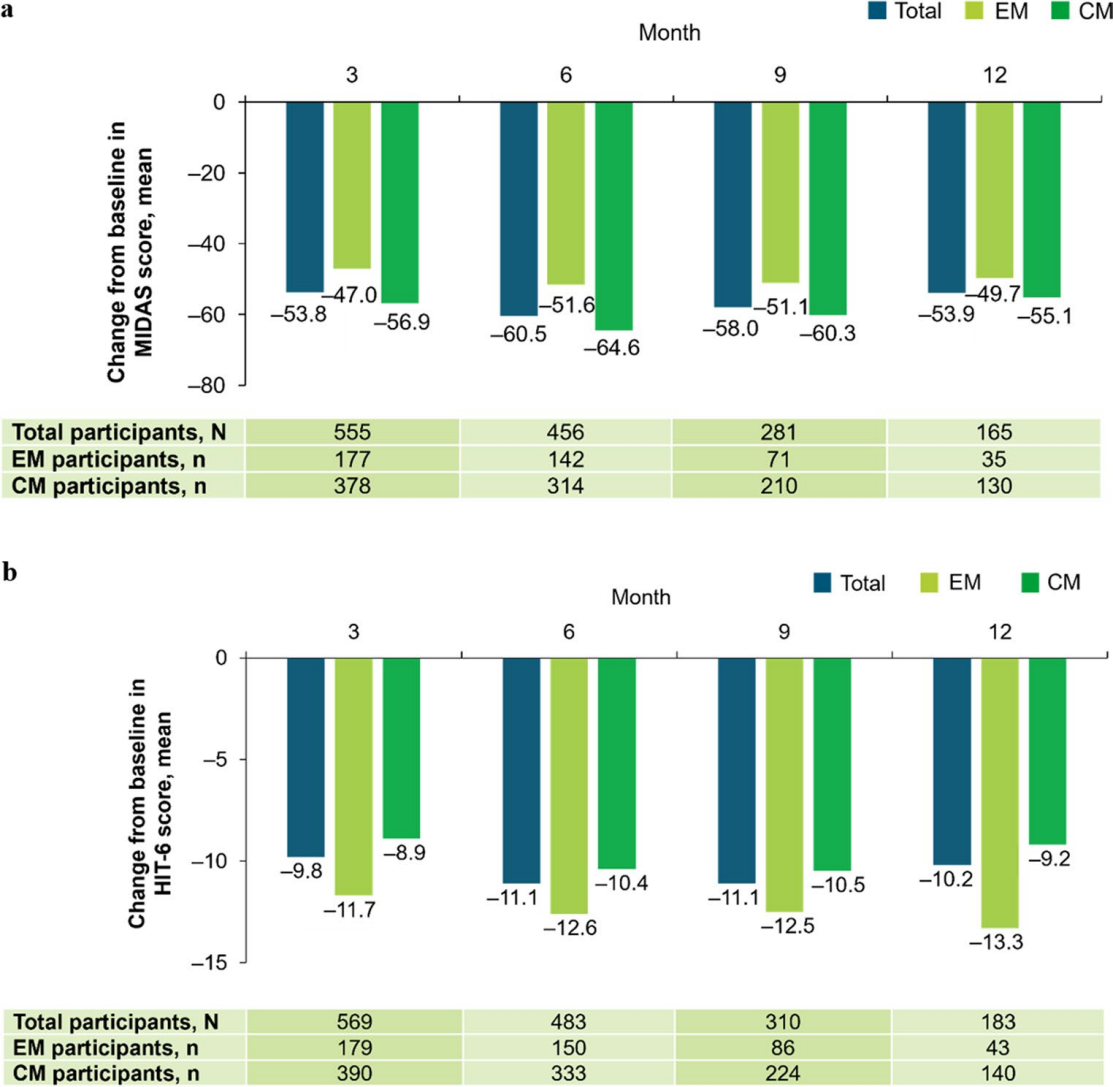
Mean change from baseline in migraine-related disability scores at Months 1, 3, 6, 9, and 12: (**a**) MIDAS score* and (**b**) HIT-6 score*. *Possible MIDAS scores ranged from 0–270 and possible HIT-6 scores ranged from 36–78, with higher scores indicating more severe disability for both scales. At data cut-off, not all data for this endpoint were available and participants with missing data have been excluded. The number of participants prematurely discontinuing this study and the number of participants that had not yet reached the relevant observation timepoint, as well as delays in data being entered into the electronic data capture system, contributed to a drop in participant numbers over time. *CM* chronic migraine, *EM* episodic migraine, *HIT-6* 6-item Headache Impact Test, *MIDAS* Migraine Disability Assessment

The mean MIDAS score for all participants was 85.6 at baseline and 34.0, 27.3, 27.0, and 23.9 at Months 3, 6, 9, and 12, respectively. The mean change from baseline in MIDAS score was ‒60.5 at Month 6 and ‒53.9 at Month 12 (Fig. 7a). The proportion of participants who reported an improvement in their MIDAS score was 50.5% at Month 3, 61.4% at Month 6, 62.3% at Month 9, and 60.0% at Month 12.

The mean HIT-6 score for all participants was 66.8 at baseline and 57.3, 55.9, 55.4, and 55.4 at Months 3, 6, 9, and 12, respectively. The mean change from baseline in HIT-6 score was ‒11.1 at Month 6 and ‒10.2 at Month 12 (Fig. 7b). The proportion of participants who reported an improvement in their HIT-6 score was 51.5% at Month 3, 58.8% at Month 6, 62.3% at Month 9, and 58.5% at Month 12.

#### Adherence and persistence with fremanezumab

**Fig. 8:**
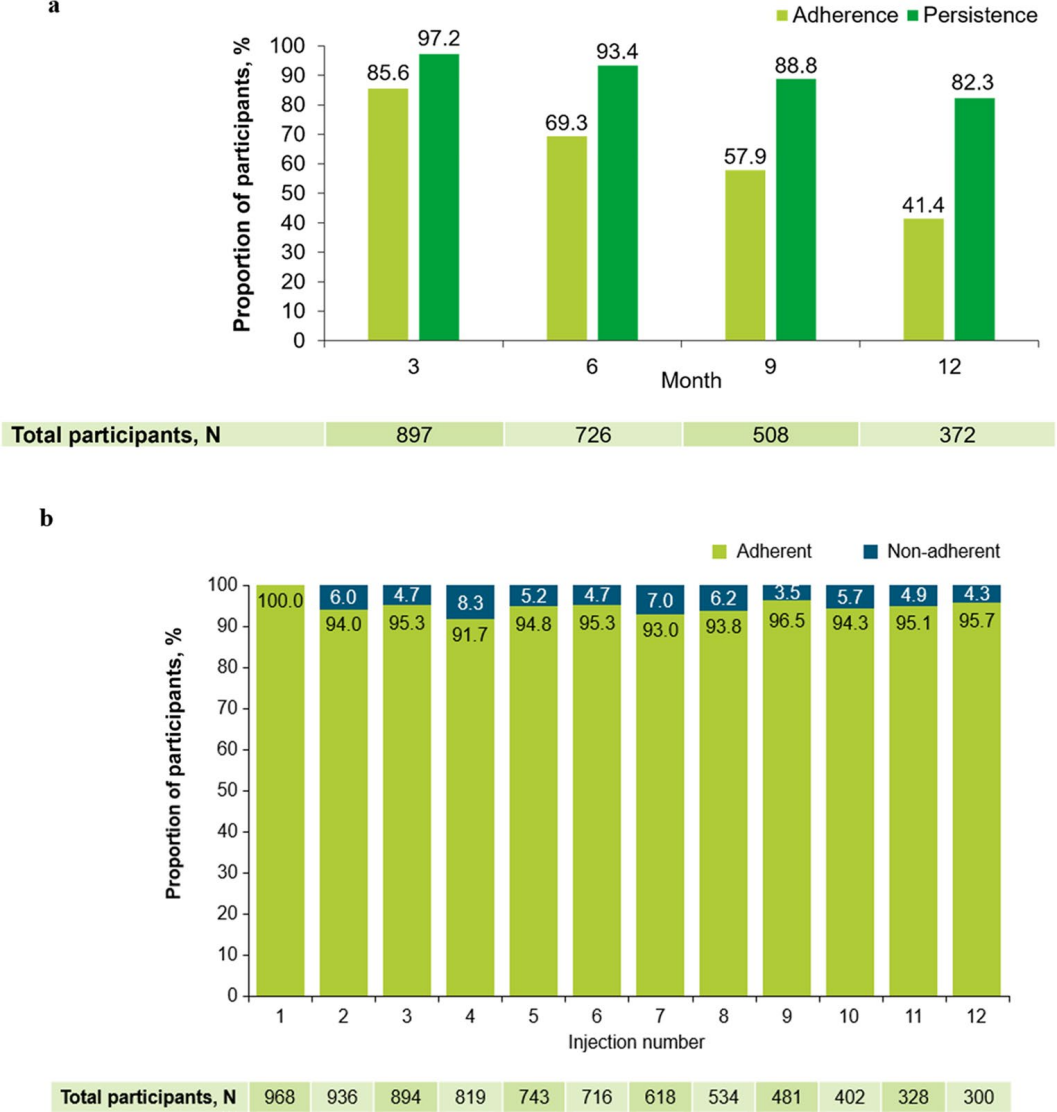
Adherence to and persistence with the fremanezumab treatment schedule at Months 1, 3, 6, 9, and 12. Participants were classified as non-adherent either (**a**) from the time of the first dose occurring outside the ± 5 days of their dosing regimen, despite adherence at subsequent timepoints, or (**b**) per injection. At data cut-off, not all data for this endpoint were available and missing data have been excluded. The number of participants prematurely discontinuing this study and the number of participants that had not yet reached the relevant observation timepoint, as well as delays in data being entered into the electronic data capture system, contributed to a drop in participant numbers over time

When participants were classified as non-adherent from the first time their prescribed dose occurred outside the ± 5 days of their dosing regimen (even if they were adherent at following timepoints), adherence to the fremanezumab treatment schedule decreased from 85.6% (n = 768) at Month 1 to 41.4% (n = 154) at Month 12 (Fig. 8a). However, when participants were classified as non-adherent if the injection timing occurred outside of ± 5 days of the scheduled dosing regimen, per injection, adherence rates remained at ≥ 90% across all injections in the first 12 months of the study (Fig. 8b). Persistence with fremanezumab treatment was largely maintained throughout the study period, with 82.3% of participants (n = 306/372 with data available) continuing treatment at Month 12 (Fig. 8a).

### Exploratory endpoints

#### Remaining headache severity and duration

**Fig. 9:**
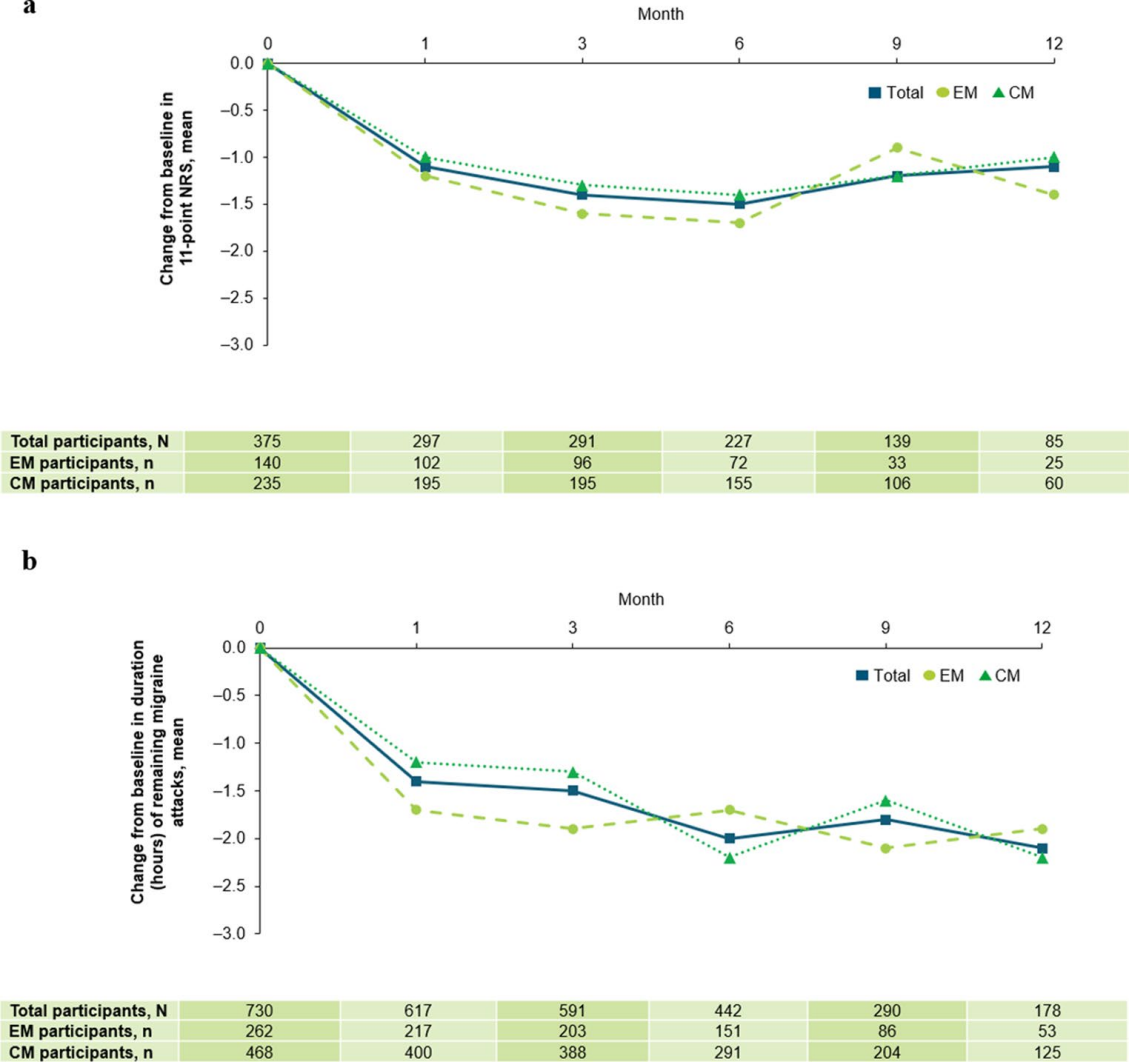
Mean change from baseline at Months 1, 3, 6, 9, and 12 in headache severity (**a**) and duration (**b**). At data cut-off, not all data for this endpoint were available and missing data have been excluded. The number of participants prematurely discontinuing this study and the number of participants that had not yet reached the relevant observation timepoint, as well as delays in data being entered into the electronic data capture system, contributed to a drop in participant numbers over time. *CM* chronic migraine, *EM* episodic migraine, *NRS* Numeric Rating Scale

The severity and duration of remaining migraine attacks decreased within 1 month after fremanezumab initiation, with reductions sustained through to Month 12 (Fig. 9a and b). In the total population, mean headache severity of migraine attacks at baseline was 6.8 on the 11-point NRS. The mean NRS values for the remaining migraine attacks decreased to 5.6, 5.4, 5.3, 5.5, and 5.1 at Months 1, 3, 6, 9, and 12, respectively. The monthly mean duration of migraine attacks was 8.4 h at baseline and decreased to 7.2, 6.8, 6.2, 6.1, and 6.1 h at Months 1, 3, 6, 9, and 12, respectively.

#### Switch participants

**Fig. 10:**
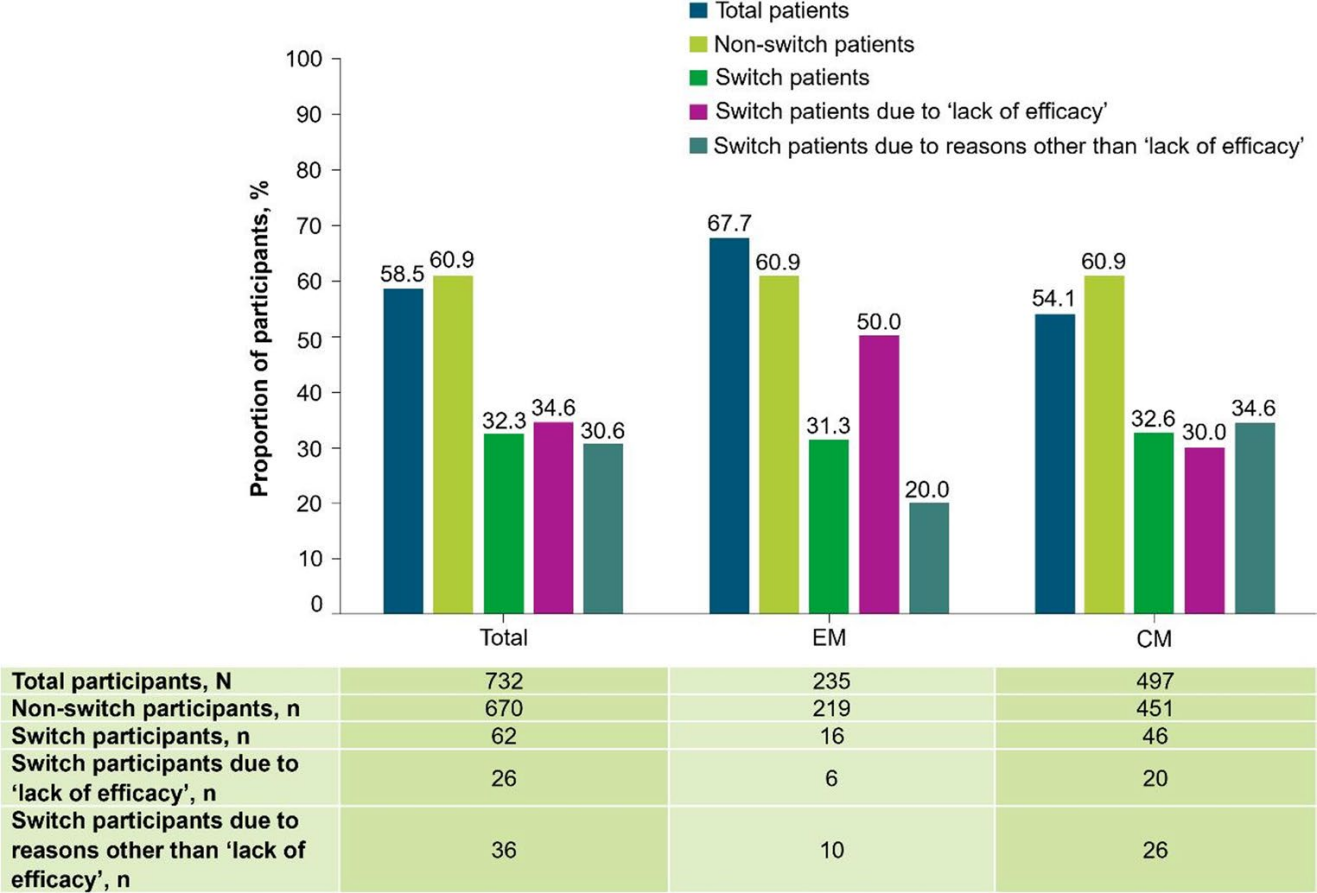
Proportion of all participants (non-switching and switching) who reached ≥ 50% reduction from baseline in MMD during the 6 months after fremanezumab initiation. Switch participants were defined as those participants switching from another CGRP pathway mAb treatment to fremanezumab. The number of participants prematurely discontinuing this study and the number of participants that had not yet reached the relevant observation timepoint, as well as delays in data being entered into the electronic data capture system, contributed to a drop in participant numbers over time. *CGRP* calcitonin gene-related peptide, *CM* chronic migraine, *EM* episodic migraine, *mAb* monoclonal antibody, *MMD* monthly migraine days

Of 732 participants with available data on the primary endpoint, 62 switched to fremanezumab from another CGRP pathway mAb (EM, n = 16; CM, n = 46). Of these, one participant had been previously treated with galcanezumab, 59 with erenumab, and two with galcanezumab and erenumab on separate occasions. Discontinuation of prior CGRP pathway mAb therapy was either due to lack of efficacy (n = 26; 41.9%), or ‘other reasons’ (n = 36; 58.1%), including AEs, withdrawal by subject, protocol deviation, pregnancy, non-compliance to study protocols, loss to follow-up, or other. During the first 6 months of fremanezumab treatment, 32.3% of the switch participants (n = 20) achieved ≥ 50% MMD reduction from baseline (Fig. 10). The proportion of switch participants that achieved ≥ 50% reduction from baseline in MMD increased from 27.7% at Month 1 to 49.2% at Month 6, and 48.9% at Month 9. Sustained reductions in MMD from baseline were observed in the switch participants from − 4.4 at Month 1 through to ‒6.3 at Month 12.

### Safety endpoints

#### Adverse events

AEs are summarized in Table 3. A total of 267 participants (23.4%) experienced ≥ 1 AE which had been classified by the investigator to be drug related. The most common drug-related AEs were general disorders and administration site conditions (n = 170; 14.9%) and gastrointestinal disorders (n = 64; 5.6%; Table 3). Serious drug-related AEs were infrequent, with only two participants (0.2%) experiencing drug hypersensitivity. AEs that led to treatment discontinuation were reported by 33 (2.9%) participants; the most common were drug ineffectiveness (n = 17; 1.5%), injection site erythema (n = 5; 0.4%), and injection site pruritus (n = 4; 0.4%).

**Table 3:** Safety analysis

	SAS (N= 1140)
Participants with ≥1 AE, n (%)	458 (40.2)
Participants with ≥1 serious AE, n (%)	37 (3.3)
Participants with ≥1 drug-related AE, n (%)^a^	267 (23.4)
Participants with drug-related serious AEs, n (%)^a^	2 (0.2)
Number of participants with AEs leading to discontinuation, n (%)	33 (2.9)
Common AEs, n (%)^b,c^	
*General disorders and administration site conditions*	187 (16.4)
Injection site erythema	83 (7.3)
Injection site pruritus	48 (4.2)
Injection site swelling	39 (3.4)
*Infections and infestations *	159 (14.0)
COVID-19	110 (9.7)
*Gastrointestinal disorders*	88 (7.7)
Constipation	46 (4.0)
Common drug-related AEs, n (%)^a,b,c^	
*General disorders and administration site conditions*	170 (14.9)
Injection site erythema	83 (7.3)
Injection site pruritus	48 (4.2)
Injection site swelling	39 (3.4)
Drug ineffective	28 (2.5)
*Gastrointestinal disorders*	64 (5.6)
Constipation	44 (3.9)

^a^Drug-related AEs that have been classified by the investigator to be related to fremanezumab treatment.

^b^Includes system organ class reported in ≥5% of the study population.

^c^Includes specific disorders reported in ≥2% of the study population.

*AE* adverse event, *COVID-19* coronavirus 2019, *SAS* safety analysis set

## Discussion

The PEARL study represents the largest and most comprehensive prospective study of fremanezumab in a real-world setting and was conducted in a diverse population comprising varied age groups, nationalities, ethnic backgrounds, comorbidities, and concomitant medications that reflect the complexity of the broad population of patients with migraine.

This third interim analysis revealed that over half of the 732 participants with available data achieved ≥ 50% reduction in MMD during the 6-month period following fremanezumab initiation. These results confirm the consistent reductions in MMD observed throughout the PEARL study period, as previously reported in the primary interim analysis and the secondary interim analyses [25, 26]. Notably, the proportion of participants achieving this clinical response in PEARL (58.5%) is higher than that observed in RCTs of fremanezumab (38–47.4%) [12–18]. This RWE is also consistent with smaller real-world studies of fremanezumab [30, 31].

The sustained achievement of the primary endpoint from the first and second interim analyses is particularly noteworthy, as long-term preventive treatment of migraine is often associated with diminished effectiveness and tolerability [3–5]. For example, real-world data on erenumab indicate a decrease in effectiveness over time. In one prospective study of erenumab, 71% of participants with CM and prior preventive treatment failure with ≥ 1 antihypertensive and ≥ 1 anticonvulsant achieved ≥ 30% reduction in MMDs at 9–12 weeks; however, this response was sustained by only 34% of participants throughout the 52-week treatment period [32]. In another, approximately half of participants (48%) with resistant CM (≥ 3 prior preventive treatment failures) were responders after 6 months of treatment, which reduced to less than one quarter of participants after 2 years [33]. In contrast, our data demonstrate persistent effectiveness with fremanezumab over both 6 and 12 months, aligning with findings from a single-center, prospective, 3-year study of fremanezumab effectiveness in patients with refractory CM [21].

Furthermore, all primary and secondary effectiveness endpoints, including reductions in acute medication use and improvements in migraine-related disability, as measured by MIDAS and HIT-6 scores, were numerically superior in the PEARL study compared with the fremanezumab RCTs [14–18]. The safety and tolerability profile observed in the PEARL study was similarly favorable. The most common drug-related AEs were injection-site reactions, and fremanezumab-related constipation was reported in 3.9% of participants, which is markedly lower than rates observed in studies of mAbs that target the CGRP receptor [32, 34–39]. Constipation was reported as a mild to severe AE in 20.0–41.3% of patients with CM in two prospective, observational studies of erenumab [32, 36], and was observed in 6.9–11.0% of patients in Phase 3 RCTs with atogepant [34, 37, 38]. Similarly, in a study of United States (US) Food and Drug Administration (FDA) Adverse Event Reporting System data, constipation was one of the most frequently reported AEs with atogepant and had the strongest signal among atogepant-related gastrointestinal AEs (reporting odd ratio [ROR] 12.85; 95% confidence interval [CI]: 11.32, 14.60) [39]. These CGRP receptor-targeting treatments also target the amylin-1 (AMY-1) receptor [40, 41], which is implicated in the regulation of gastric motility [42]. Therefore, these effects on gastric motility, including constipation, may be mediated by the interaction of CGRP receptor-targeting treatments blocking the activity of the AMY-1 receptor.

Exploratory endpoints included in this analysis further indicate that fremanezumab not only reduces the frequency of migraine attacks, but also decreases their severity and duration, thereby enhancing patients’ overall quality of life and functional ability. While the clinical success of migraine preventive treatment is typically measured by a reduction in MMD, for some participants, the severity and duration, rather than the frequency of migraine attacks, have a greater impact on their daily lives [43]. Increased duration and severity of migraine has been shown to be associated with increased migraine-related disability and psychological comorbidity, and decreased quality of life and workplace productivity [1]. Thus, reducing headache severity and duration are important determinants of restoring functional ability and quality of life for participants with migraine. Within 1 month of fremanezumab initiation, participants experienced a reduction in the mean duration and severity of remaining migraine attacks, which was sustained up to 12 months.

Poor adherence can lead to decreased health outcomes, increased hospitalizations, and higher healthcare costs [44]. The adherence and persistence of patients to non-specific oral preventive migraine medications are variable, but generally low [5, 45]. Although adherence to monthly erenumab was found to be higher than adherence to traditional oral preventive migraine therapies, overall adherence was still suboptimal (ranging from 31–42% over 6 months) in a retrospective US claims database study [45]. Data regarding the adherence and persistence of participants to fremanezumab treatment in the PEARL study are reported for the first time in the present interim analysis. Our data demonstrated that participants receiving fremanezumab exhibited very high persistence, with the majority remaining on treatment at Month 12. Adherence to fremanezumab was reduced over time as participants were defined as “non-adherent” from the time of their prescribed dose occurring outside the ± 5 days of their dose regimen, even if they were adherent at following timepoints. Since this may not accurately reflect any potential improvement in adherence over time, a more accurate calculation method was developed to assess adherence to fremanezumab from an alternative perspective. When participants were classified as non-adherent from the time of the first dose occurring outside of ± 5 days of the scheduled dosing regimen, per injection, results indicated that adherence rates remained at ≥ 90% from injections 1–12 inclusive. Adherence to fremanezumab treatment has been shown previously to be high in retrospective US real-world studies, with quarterly dosing associated with higher rates of adherence compared with monthly dosing [46, 47].

The PEARL study design has some relevant limitations. First, the outcomes reported within the participant headache diaries relied on the accuracy of the recorder, and thus, were subject to human error and participant diligence and subjectivity. In some instances, data were missing, resulting in reduced sample sizes at each timepoint, therefore the data collections and comparisons taken at Month 6 and beyond should be interpreted with caution. This is an inherent risk of real-world studies, where the occurrence of missing data is generally more common than in RCTs [48]. Despite these limitations, the PEARL study remains the largest prospective study of fremanezumab, with a greater number of participants than pivotal RCTs. As the study progresses, more data will become available, which will include a larger and more representative population of participants, ensuring more robust evidence.

## Conclusions

Overall, the findings from this third interim analysis of the PEARL study demonstrate the sustained effectiveness of fremanezumab with regards to reducing MMD, acute medication use, and migraine-related disability over 12 months in a large real-world population. High treatment persistence and adherence, coupled with a favorable safety profile, support the continued use of fremanezumab as a long-term preventive treatment for migraine. These results, which align with previous interim analyses and RCTs, provide robust evidence for the role of fremanezumab in improving clinical outcomes for patients with migraine.

## Data Availability

Qualified researchers may request access to patient level data and related study documents including the study protocol and the statistical analysis plan. Requests will be assessed for scientific merit, product approval status, and conflicts of interest. If the request is approved, patient level data will be de-identified and study documents will be redacted to protect the privacy of trial participants and to protect commercially confidential information. Please email USMedInfo@tevapharm.com to make your request.
